# MicroRNA‐214‐3p modified tetrahedral framework nucleic acids target survivin to induce tumour cell apoptosis

**DOI:** 10.1111/cpr.12708

**Published:** 2019-10-23

**Authors:** Songhang Li, Yue Sun, Taoran Tian, Xin Qin, Shiyu Lin, Tao Zhang, Qi Zhang, Mi Zhou, Xiaolin Zhang, Yi Zhou, Hu Zhao, Bofeng Zhu, Xiaoxiao Cai

**Affiliations:** ^1^ State Key Laboratory of Oral Diseases National Clinical Research Center for Oral Diseases West China Hospital of Stomatology Sichuan University Chengdu China; ^2^ College of Basic Medicine Chengdu University of Traditional Chinese Medicine Chengdu China; ^3^ Department of Restorative Sciences College of Dentistry Texas A&M University Dallas TX USA; ^4^ Key Laboratory of Shaanxi Province for Craniofacial Precision Medicine Research College of Stomatology Xi’an Jiaotong University Xi’an China; ^5^ Clinical Research Center of Shaanxi Province for Dental and Maxillofacial Diseases College of Stomatology Xi’an Jiaotong University Xi’an China; ^6^ Department of Forensic Genetics School of Forensic Medicine Southern Medical University Guangzhou China

**Keywords:** apoptosis, caspase3, microRNA, survivin, tetrahedral framework nucleic acids

## Abstract

**Objectives:**

Due to the instability of microRNAs, the applications of microRNA are currently limited. Thus, we utilized tetrahedral framework nucleic acids and a targeted microRNAs to form a stable nanocomposite to explore whether this nanocomposite can promote apoptosis of tumour cells.

**Materials and methods:**

In our study, the survivin gene, which is expressed only in tumour cells and embryonic cells, was selected as the target gene; miRNA‐214‐3p, which can reduce the expression of survivin, was modified onto tetrahedral framework nucleic acid, thereby producing a reduction in the expression of survivin upon intracellular delivery and eventually leading to tumour cell apoptosis.

**Results:**

By comparing the stability of microRNAs with that of microRNA‐tetrahedral framework nucleic acid, we proved the superiority of this carrier system. The results of flow cytometry showed that after treated with this complex, the ratio of A549 cells in both late and early period of apoptosis in miRNA‐214‐3p‐tetrahedral framework nucleic acid group had doubled and the cell cycle in the G2‐M phase had declined. The decrease in the expression of anti‐apoptotic protein and the increase in the expression of pro‐apoptotic protein indicate that the ability of this complex to function in cells also makes it attractive as a new targeted therapy for cancer.

**Conclusion:**

The unique expression of survivin in tumour cells and embryonic cells makes microRNA‐tetrahedral framework nucleic acid a new targeted therapy. In addition, due to the functional diversity of microRNAs, this delivery system approach can be applied to a wide variety of fields, such as targeted therapy and tissue regeneration.

## 1 INTRODUCTION

DNA nanotechnology has developed rapidly in the past 30 years.^1^ Owing to the relative ease with which DNA can be edited, scholars have used DNA to assemble nanomaterials with different spatial structures and morphologies.^2^, ^3^, ^4^, ^5^, ^6^, ^7^ In addition, many studies have concluded that these nanomaterials have good biocompatibility.^8^ Based on these advantages, DNA nanomaterials have been applied in many fields, such as biotherapy, targeted transportation and in vivo imaging.^9^, ^10^, ^11^ Compared with other types of nanomaterials, tetrahedral framework nucleic acids (tFNAs) have become a research hotspot in recent years due to their unique merits.^8^, ^12^ tFNAs can be taken up by cells via caveolin‐mediated endo‐cytosis, and tFNAs possess better serum stability than other nanomaterials with non‐arbitrary spatial structures.^13^, ^14^ At the same time, tFNAs have lower biological toxicity, which greatly broadens the application prospects of tFNAs.^15^, ^16^, ^17^, ^18^ In previous studies from our group, we incubated tFNAs with chemotherapeutic drugs or modified tFNAs with adapters, ultimately using the permeability of tFNAs to penetrate the cell membrane into cells. These conclusions also indicate that tFNAs have great potential in the field of drug delivery.^16^, ^19^

MicroRNAs (miRNAs) are endogenous, small noncoding RNAs that are produced from stem‐loop regions of pri‐miRNAs.^20^ It has been proven that miRNAs can interact with human RNA and that a single mRNA can be regulated by one or several miRNAs, which implies that miRNAs affect almost all developmental processes and diseases.^21^ MiRNAs are extremely abundant in the human body and form an intricate regulatory network in human cells.^22^ Furthermore, due to the key role of miRNAs in tumour cells, miRNAs are gradually being applied to the field of antitumour therapy.^20^, ^23^ However, because of the instability of miRNAs, therapeutically relevant applications of miRNAs are extremely limited.^24^ The current transfection methods of liposomes and lentiviruses can also achieve better transfection efficiency, but due to the cytotoxicity of liposomes and lentiviruses, these two methods cannot be widely used in the field of targeted therapy. While tFNAs have good biocompatibility and can overcome this disadvantage when carrying. Therefore, we linked tFNAs to miRNAs, allowing tFNAs to serve as vectors for miRNAs and utilizing the uptake potential of tFNAs to carry miRNAs into cells. The use of this delivery method can also overcome the instability of miRNAs, thereby increasing their intracellular efficiency. Moreover, this is the first time that miRNAs have been bound to tFNAs to produce a therapeutic effect.^12^

**Figure 1:**
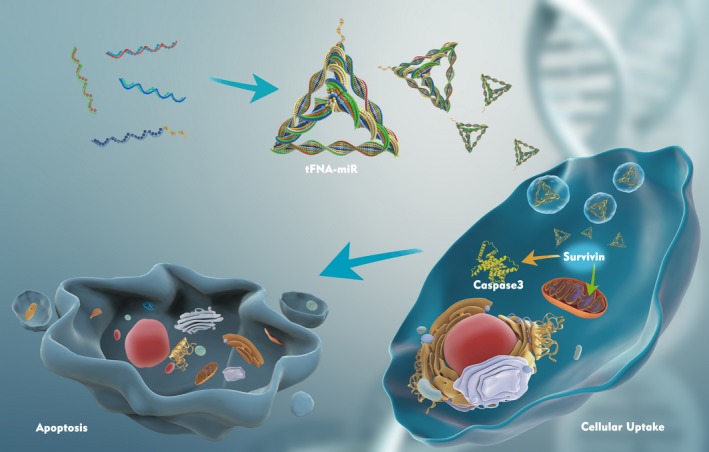
Schematic illustration showing the preparation of tFNA‐miR and the mechanism of tFNA‐miR inducing apoptosis of A549 cells

Survivin, a unique inhibitor of apoptosis (IAP) protein, has been shown to be overexpressed in malignancies compared to normal adult tissue.^25^ As this protein is overexpressed in tumour cells and embryonic cells but is undetectable in normal tissues, it is an attractive candidate for tumour‐targeted therapy.^26^ MiRNA‐214‐3p (miR‐214‐3p) can bind to survivin mRNA, which reduces the stability of survivin mRNA. Consequently, the expression of survivin protein decreases, eventually leading to tumour cell apoptosis.^27^ In this study, we modified miR‐214‐3p to complex with tFNAs (tFNAs‐miR‐214‐3p) and observed the ability of miR‐214‐3p to induce apoptosis of tumour cells (Figure 1).^28^ In addition, we validated the stability of tFNAs‐miR‐214‐3p in a variety of enzymatic environments by assaying serum stability.

## 2 MATERIALS AND METHODS

### 2.1 Synthesis of tFNAs and tFNAs‐miR‐214‐3p

Generally, three ssDNAs (S1, S2, S4) and one ssDNA modified with microRNA (S3‐miR‐214‐3p), synthesized by Takara (Dalian), were mixed with equimolar quantities into TM buffer (50 mmol/L MgCl2.6H2O, 10 mmol/L Tris‐HCl, pH = 8.0) and then were denatured at 95°C for 10 minutes prior to cooling to 4°C for 30 minutes. All DNA sequences are listed in Table 1.

**Table 1:** Base sequence of each ssDNA (capital letters denote DNA monomers, and lowercase letters denote RNA monomers)

ssDNA	Sequences (from 5′ to 3′)
S1	ATTTATCACCCGCCATAGTAGACGTATCACCAGGCAGTTGAGACGAAC ATTCCTAAGTCTGAA
S2	ACATGCGAGGGTCCAATACCGACGATTACAGCTTGCTACACGATTCAG ACTTAGGAATGTTCG
S3	ACTACTATGGCGGGTGATAAAACGTGTAGCAAGCTGTAATCGACGGG AAGAGCATGCCCATCC
S4	ACGGTATTGGACCCTCGCATGACTCAACTGCCTGGTGATACGAGGATG GGCATGCTCTTCCCG
S3‐miR‐214‐3p	acagcaggcacagacaggcaguTTTTTACTACTATGGCGGGTGATAAAACGTGTA GCAAGCTGTAATCGACGGGAAGAGCATGCCCATCC

### 2.2 Characterization of tFNAs and tFNAs‐miR‐214‐3p

We used 8% SDS‐PAGE and capillary gel electrophoresis instrument (Qsep100, Bioptic) to verify the successful synthesis of tFNAs and tFNAs‐miR‐214‐3p. TEM (Libra200, Zeiss) was used to detect the morphological structure of tFNAs and tFNAs‐miR‐214‐3p. The ζ‐potential of tFNAs and tFNAs‐miR‐214‐3p was evaluated by DLS on a Zeta‐sizer Nano ZS90 (Malvern Instrument Ltd).

### 2.3 Cell culture

Non‐small cell lung cancer cell lines A549 were obtained from our own group. A549 cells were cultured in Dulbecco's modified Eagle's medium/nutrient mixture F‐12 (DMEM/F12, HyClone) mixed with penicillin/streptomycin solution (HyClone) and 10% FBS (Corning) in a humidified incubator (37°C). When the density of A549 increased to 80%‐90%, the A549 were passaged with trypsin.

### 2.4 Cellular uptake of tFNAs‐miR‐214‐3p

To observe the cellular uptake of tFNAs‐miR‐214‐3p, S1 was labelled with Cy5. Following the synthesis process described above, we obtained Cy5‐tFNAs‐miR‐214‐3p, which can be visualized with a confocal laser microscope (TCS SP8, Leica). A549 were sowed in 24‐well plates with DMEM/F12 for 24 hours and then starved for 2 hours. Subsequently, we replaced the previous cell culture medium with FBS‐free media supplemented with Cy5‐tFNAs‐miR‐214‐3p for 8 hours. A549 were rinsed with PBS (HyClone) and then fixed with 4% cold polyoxymethylene solution for 15 minutes. Afterwards, A549 were rinsed again and then stained with DAPI and phalloidin for 10 minutes and 20 minutes, respectively. Finally, we observed the cellular uptake with the confocal laser microscope and captured the images.

### 2.5 Stability of tFNAs‐miR‐214‐3p

For sake of proving that the combination of microRNAs and tFNAs can enhance the stability of microRNAs, we mixed FBS at a concentration of 1% in tFNAs (1000 nmol/L) and then incubated the mixture in a humidified incubator. After incubating miR‐214‐3p for 1, 5 and 30 minutes, we used a capillary gel electrophoresis instrument (Bioptic) to observe the changes in RFU. When incubating tFNAs, miR‐214‐3p, tFNAs‐miR‐214‐3p with 10% FBS for 24 hours, we used 1% agarose gel electrophoresis to confirm the stability of tFNAs‐miR‐214‐3p.^29^

### 2.6 CCK‐8 assay

A549 were seeded in a 96‐well plate (4000 per well), followed by sequentially preincubated in 10% FBS cell culture medium for 24 hours, 5% FBS cell culture medium for 12 hours, 1% FBS cell culture medium for 10 hours and 0% FBS cell culture medium for 2 hours. Afterwards, tFNAs‐miR‐214‐3p was added at test concentrations of 150 and 250 nmol/L for 72 hours. Finally, CCK‐8 (Dojindo) was added into the plate and incubated for 1 hours. The optical density (OD) was recorded at 450 nm.

### 2.7 Apoptosis assay

A549 cells were transferred onto coverslips and treated with tFNAs and tFNAs‐miR‐214‐3p for 72 hours. Afterwards, we used phase‐contrast microscopy (DMi8; Leica) to observe the changes in cell morphology and number. Subsequently, cells on the coverslip were rinsed twice and dyed with dyeing buffer (BestBio). The coverslips were treated with 5 μL acridine orange (AO; BestBio) and 5 μL propidium iodide (PI; BestBio) for 20 minutes at 4°C, respectively. Finally, after washing twice with PBS per 5 minutes, a fluorescence microscope (DMi8; Leica) was utilized to capture the images.

### 2.8 Quantitative real‐time polymerase chain reaction

The cells were lysed with TRIzol reagent (Life Technologies), and the total RNA was purified with RNeasy Plus Mini Kit (BioTeke). After reverse transcription, the cDNA was synthesized. According to the protocol of manufacturer, cDNA was amplified by Bio‐Rad CFX96 detection system (Bio‐Rad).

### 2.9 Flow cytometry for cell apoptosis analysis

We divided the cells into three groups. The treatment group was treated with 150 nmol/L tFNAs‐miR‐214‐3p in 0% FBS DMEM/F12, the control group was treated with 150 nmol/L tFNAs in 0% FBS DMEM/F12, and the blank group was treated with the same volume of TM buffer to avoid the error caused by diluting the medium. After 72 hours, cells were collected by trypsin solution without enediaminetetraacetic acid (Solarbio) and washed with PBS twice. To each group, we added 500 μL of binding buffer (KeyGen Biotech) followed by 5 μL of Annexin V‐FITC and 5 μL of propidium iodide (PI; KeyGen Biotech) that were incubated for 15 minutes and 5 minutes, respectively. Ultimately, the compounds were measured by a flow cytometer (FC500 Beckman).

### 2.10 Flow cytometry for cell cycle analysis

Samples were processed into the same three groups as described above. After 72 hours of treatment, we fixed the samples with ice‐cold ethanol overnight. Then, we added 500 μL of RNase (KeyGen Biotech) to the samples at 37°C for 30 minutes and mixed 400 μL of propidium iodide (PI; KeyGen Biotech) at 4°C for 30 minutes. Finally, the samples were detected by the flow cytometer (FC500 Beckman).

### 2.11 Immunofluorescence assay

A549 cells were transferred into coverslip and treated with the same three groups as mentioned above for 72 hours. Then, cells were fixed with 4% paraformaldehyde for 15 minutes and permeabilized by 0.5% Triton X‐100 for 10 minutes. Subsequently, the samples were incubated with 5% goat serum for 1 hour then with diluted primary antibodies, Caspase3 (1:500; ab184787), Bax (1:500; ab53154) and Bcl‐2 (1:250; ab182858) (all from Abcam), at 4°C overnight. Subsequently, we rinsed thrice and incubated with secondary antibody (1:500; Invitrogen) at 37°C for 1 hour, which was followed by DAPI and phalloidin application to stain with nucleus and cytoskeleton, respectively. Finally, the samples were soaked in 10% (v/v) glycerol.

### 2.12 Western blot

We used an extraction kit (KeyGen Biotech) to extract the total protein. The protein samples were added to 5× loading buffer (Beyotime) by volume, and the samples were placed in boiling water for 5 minutes and then stored at −20°C. Protein samples were separated by PAGE electrophoresis and then transferred to polyvinylidene difluoride (PVDF) membranes. After blocking with 5% bovine serum albumin (BSA) for 1 hour, the membranes were soaked with Caspase3 (1:2000; ab184787), Bax (1:1000; ab53154) and Bcl‐2 (1:2000; ab182858) (all from Abcam) primary antibodies and stored at 4°C overnight. Subsequently, the PVDF were rinsed thrice with Tris‐buffered saline with Tween 20 (TBST) and incubated with secondary horseradish peroxidase‐conjugated goat anti‐rabbit IgG secondary antibody (SAB) for 1 hour. Finally, the membranes were exposed by using an enhanced chemiluminescence detection system (Bio‐Rad). We used the ImageJ v1.52a software to analyse the grey value of each membrane. In addition, we set GAPDH as an internal control.

### 2.13 Statistical analysis

Quantitative data are presented as the mean ± standard deviation (SD), and comparisons between groups were analysed by one‐way analyses of variance (ANOVA) or *t* test. *P* values were considered statistically significant when <.05. All the experimental results were statistically analysed using GraphPad Prism v8.0.2 (GraphPad).

## 3 RESULTS

### 3.1 Preparation and characterization of tFNAs and tFNAs‐miR‐214‐3p

**Figure 2:**
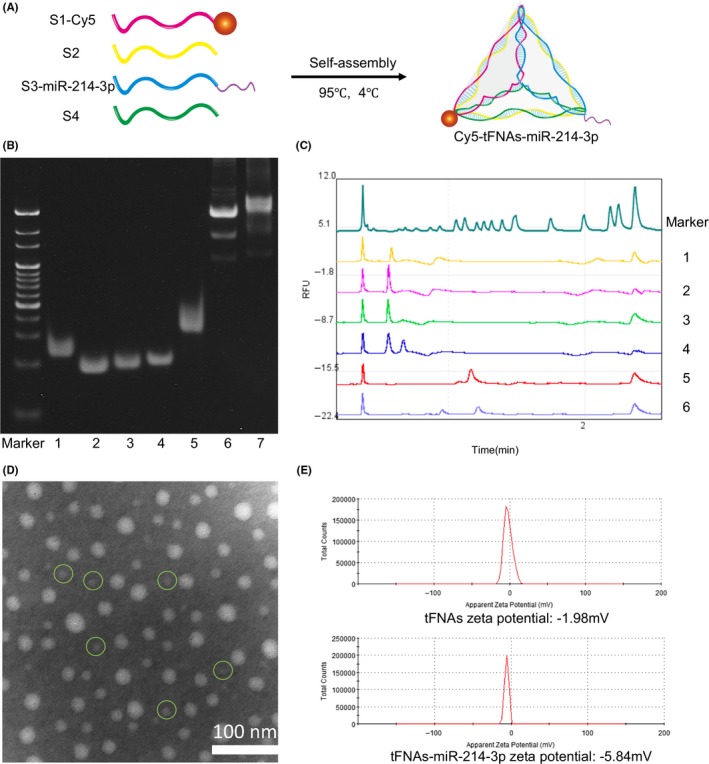
Successful preparation of tFNAs and tFNAs‐miR‐214‐3p. A, Diagrammatic sketch of Cy5‐tFNAs‐miR‐214‐3p. B, Analysis by 8% PAGE. (1: S1; 2: S2; 3: S3; 4: S4; 5: S3‐miR‐214‐3p; 6: tFNAs; 7: tFNAs‐miR‐214‐3p). C, Proof of the successful synthesis of tFNAs and tFNAs‐miR‐214‐3p by capillary gel electrophoresis. (1: S1; 2: S2; 3: S3; 4: S4; 5: tFNAs; 6: tFNAs‐miR‐214‐3p). D, TEM analysis of tFNAs‐miR‐214‐3p. E, Zeta potential graphs of tFNAs and tFNAs‐miR‐214‐3p

Conventionally, tFNAs are composed of four isometric single‐strand DNAs (ssDNA), and the tumour‐targeting miR‐214‐3p is modified to a vertex of tFNAs. MiR‐214‐3p was modified to the 5’ end of S3 to form a new single strand (S5) (Table 1); then, tFNAs‐miR‐214‐3p was synthesized following the same procedure as tFNAs (Figure 2A).^12^, ^30^ To prove that we successfully synthesized tFNAs‐miR‐214‐3p, polyacrylamide gel electrophoresis (PAGE) was utilized. Figure 2B shows the position of S1, S2, S3, S4, S5, tFNAs and tFNAs‐miR‐214‐3p; it is obvious that S5 is significantly longer than the remaining four ssDNAs, and the migration rate of tFNAs‐miR‐214‐3p is slower than that of tFNA, which demonstrated the successful synthesis of tFNA and tFNAs‐miR‐214‐3p. To further verify the successful synthesis of the nanomaterials, capillary gel electrophoresis was carried out, and the obtained relative fluorescence units (RFU) results confirmed the PAGE results (Figure 2C). As we observed from transmission electron microscope (TEM) imaging, the shape of the tetrahedron structure could be recognized, and the size of the nanomaterial was found to be approximately 20 nm (Figure 2D, green circle). While some polymerization occurred during synthesis, the agglomeration had no effect on the function of tFNAs compared with the monomers.^15^, ^31^, ^32^ Since the nucleic acid molecules are negatively charged, we observed that the ζ‐potential of tFNAs‐miR‐214‐3p was lower than that of tFNAs (Figure 2E) via dynamic light scattering (DLS).^17^

### 3.2 Cellular uptake of tFNAs‐miR‐214‐3p

**Figure 3:**
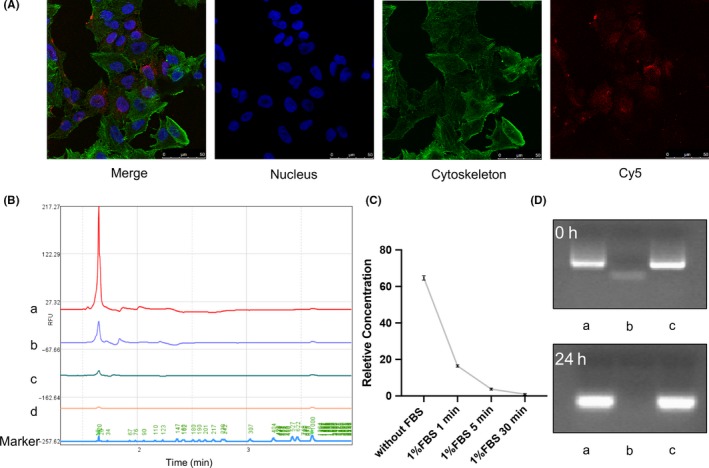
Cellular uptake of tFNAs‐miR‐214‐3p and stability of tFNAs‐miR‐214‐3p in enzymatic environment. A, Cellular uptake of Cy5‐tFNAs‐miR‐214‐3p by confocal microscope (nucleus: blue; cytoskeleton: green; Cy5: red). Scale bars are 25 μm. B, Relative fluorescence units of miR‐214‐3p with 1% FBS. (a: without FBS; b: 1% FBS, 1min; c: 1% FBS, 5min; d: 1% FBS, 30 min). C, Relative concentration of miR‐214‐3p with 1% FBS. D, Analysis by 1% agarose gel electrophoresis after treated with 10% FBS for 24 h (a: tFNAs; b: miR‐214‐3p; c: tFNAs‐miR‐214‐3p)

To track the cellular uptake of tFNAs‐miR‐214‐3p, S1 was labelled with Cy5 (Cy5‐S1).^13^ We synthesized Cy5‐tFNAs‐miR‐214‐3p with Cy5‐S1 and observed cellular uptake with confocal laser microscope after 8 hours of incubation. We observed that the fluorescence intensity (Cy5) was mainly concentrated in the cytoplasm of A549 cells, and no fluorescence was found outside the cell membrane, which demonstrated that Cy5‐tFNAs‐miR‐214‐3p successfully penetrated cells (Figure 3A).^33^, ^34^

### 3.3 Stability of tFNAs‐miR‐214‐3p in an enzymatic environment

To further demonstrate the stability advantage of tFNAs‐miR‐214‐3p over miR‐214‐3p, we attempted to verify its stability in an enzymatic environment. Foetal bovine serum (FBS) contains a variety of enzymes, so we chose to use FBS to construct this more complex enzyme environment. The stability of the smaller fragment of miR‐214‐3p was determined by capillary electrophoresis experiments (Figure 3B). By measuring the RFU, we found that after adding 1% (v/v) FBS, a large amount of degradation occurred in miR‐214‐3p within 1 minutes, and the RFU value of miR‐214‐3p could not be detected after 30 minutes (Figure 3C).

After synthesizing the tFNAs, miR‐214‐3p and tFNAs‐miR‐214‐3p were added at the same concentration and incubated. To confirm the high stability of tFNAs‐miR‐214‐3p in a complex enzyme environment, we added 10% (v/v) FBS to the synthesized samples and ran 1% agarose gel electrophoresis on them after incubating for 0 hour and 24 hours.^29^ We found that under the condition of 10% FBS, tFNAs and tFNAs‐miR‐214‐3p were generally stable for 24 hours, although the bands were slightly diffused. However, the presence miR‐214‐3p alone could not be observed after 24 hours (Figure 3D). This also implied that tFNAs‐miR‐214‐3p could be stably maintained for 24 hours or more in a high‐enzyme environment.

### 3.4 The role of tFNAs‐miR‐214‐3p in cell viability

**Figure 4:**
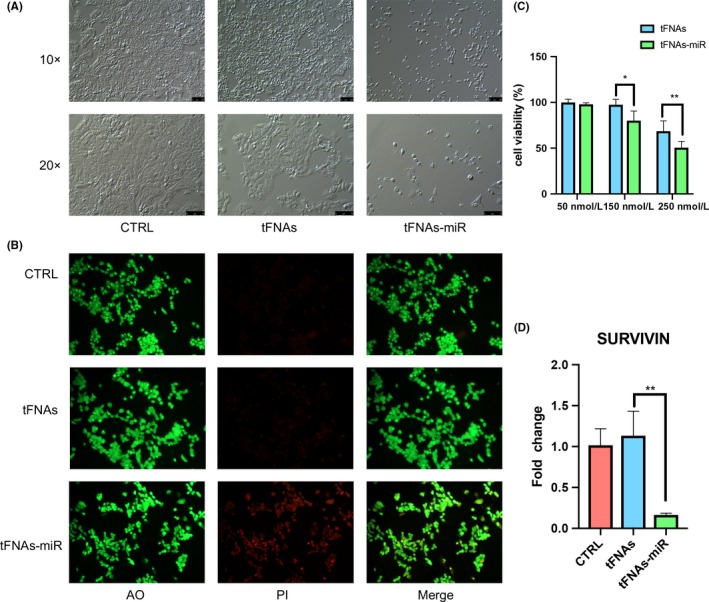
The addition of tFNAs‐miR‐214‐3p promotes the apoptosis of A549 cells. Cells are treated for 72 h. A, Images of A549 cells taken by phase‐contrast microscope. 10× and 20× scale bars are 100 μm and 75 μm, respectively. B, Using AO/PI staining and observation by fluorescence microscopy. C, CCK‐8 assay analyse the cytotoxicity with different concentrations (50 nmol/L, 150 nmol/L and 250 nmol/L). Data are presented as mean ± standard deviation (SD) (n = 4). Statistical analysis: *P < .05, **P < .01. D, qPCR analysis of survivin. Data are presented as mean ± standard deviation (SD) (n = 4). Statistical analysis: **P < .01

To prove tFNAs‐miR‐214‐3p could interact with the mRNA of survivin to induce apoptosis of tumour cells, we selected non‐small cell lung cancer (NSCLC) cells (A549) with high expression of survivin as the therapeutic target.^26^, ^28^, ^35^ The cytotoxicity of tFNAs‐miR‐214‐3p was assessed through a cell counting kit‐8 (CCK‐8) assay at concentrations of 50, 125 and 150 nmol/L. After a 72 hours treatment with tFNAs‐miR‐214‐3p, a significant decrease in cell viability occurred at both 150 nmol/L and 250 nmol/L, but since the 250 nmol/L tFNAs (no‐load vehicle) produced toxic effects on the cells and the 150 nmol/L tFNAs showed no cytotoxicity to the cells, 150 nmol/L was chosen as the ideal concentration for this experiment (Figure 4C). The morphology and number of cells at this time point were observed by phase‐contrast microscopy. Figure 4A shows that the quantity of A549 cells decreased and that the cells appeared smaller morphologically, indicating that some cells have undergone apoptosis after treatment. However, the cells in the tFNA group were basically identical to the cells in the control group, which again proved that tFNA did not produce an apoptosis‐promoting effect on A549. The A549 cells were dyed with acridine orange/propidium iodide (AO/PI) and observed by fluorescence microscopy; it was found that the tFNAs‐miR‐214‐3p group showed more pronounced red fluorescence, which also confirmed that the cells in this group were in different degrees of apoptosis (Figure 4B). The presence of red fluorescence was hardly detected in control group and tFNAs group, which demonstrated that tFNAs have no toxic effects on cells.

### 3.5 Effect of tFNAs‐miR‐214‐3p on cell apoptosis and cycle

**Figure 5:**
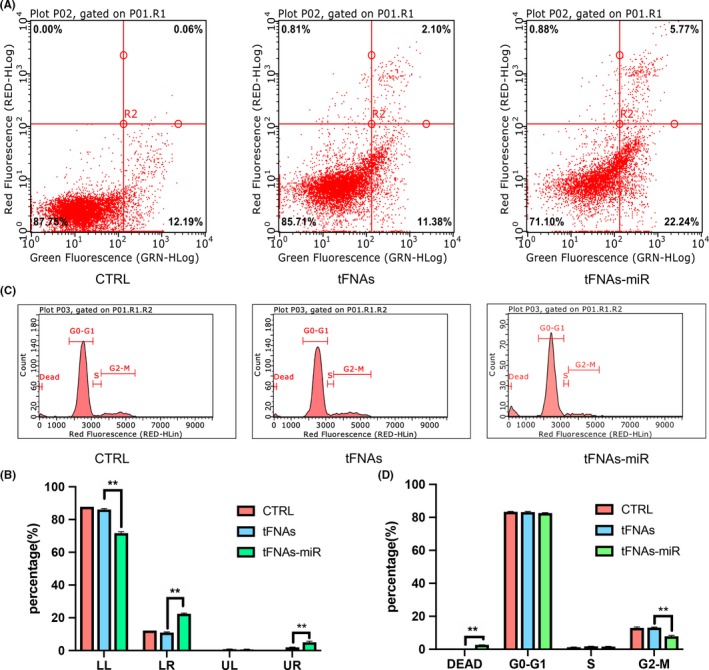
The analysis of cell apoptosis and cell cycle by flow cytometry. A, Flow cytometry examination of cell apoptosis upon exposure to tFNAs‐miR‐214‐3p. B, Data analysis of cell apoptosis by flow cytometry. Data are presented as mean ± standard deviation (SD) (n = 4). Statistical analysis: **P < .01. C, The changes of cell cycle measured by flow cytometry. D, Data analysis of cell cycle by flow cytometry. Data are presented as mean ± standard deviation (SD) (n = 4). Statistical analysis: **P < .01

In mammalian cells, phosphatidylserine (PS) merely locates in the inner phospholipid bilayer of cytomembrane, whereas in the prophase of apoptosis, PS in cytomembrane turns from medial to lateral. Annexin V is capable of binding to phosphatidylserine. Accordingly, Annexin V is used as one of the susceptible markers for testing early apoptotic cells. PI is a nucleic acid dyestuff that does not permeate the undamaged cytomembrane; for cells in a late stage of apoptosis, PI can penetrate the cytomembrane to dye the nucleus. Consequently, by matching Annexin V with PI, cells in diverse apoptotic phase can be differentiated.^36^ Using this method, we tested each group of cells and found that after 72 hours, and the ratio of A549 cells in both late and early period of apoptosis in tFNAs‐miR‐214‐3p group had doubled (Figure 5A). This suggests that microRNAs play a role in promoting apoptosis in cells (Figure 5B).

Some scholars have reported that inhibition of survivin mRNA expression will lead to a decrease in G2‐M phase cells, so we used flow cytometry to detect each group.^37^ Similarly, after 72 hours of processing, A549 cells were stained with PI, and it was found that the quantity of cells in the G2‐M phase had declined and the quantity of dead cells had increased (Figure 5C). Together, these data indicate that tFNAs‐miR‐214‐3p act on the survivin mRNA and modify the cell cycle (Figure 5D).

### 3.6 Effect of tFNAs‐miR‐214‐3p on the intrinsic pathway

The mammalian mitochondrial apoptotic pathway is mainly regulated by two protein families: the BCL‐2 family and IAP family. The BCL‐2 family regulates mitochondrial membrane potential and membrane permeability by increasing (BAX, BAK and BAD) or decreasing (BCL‐2 and BCL‐XL) BCL‐2 protein expression, thereby regulating the release of apoptotic factors (Cyt C, AIF, SMAC/DIABLO, HTRA2/OMI and ENDOG) into the mitochondria. The IAP proteins (Survivin, XIAP, cIAP‐1 and cIAP‐2) regulate the downstream of the mitochondrial apoptotic pathway by inhibiting the activation of caspase3 in the caspase family, which is considered an effector or executioner caspase. In summary, in the mitochondrial apoptosis pathway, there is downregulation of BCL‐2 and survivin and upregulation of BAX and caspase3.^25^, ^38^, ^39^, ^40^

**Figure 6:**
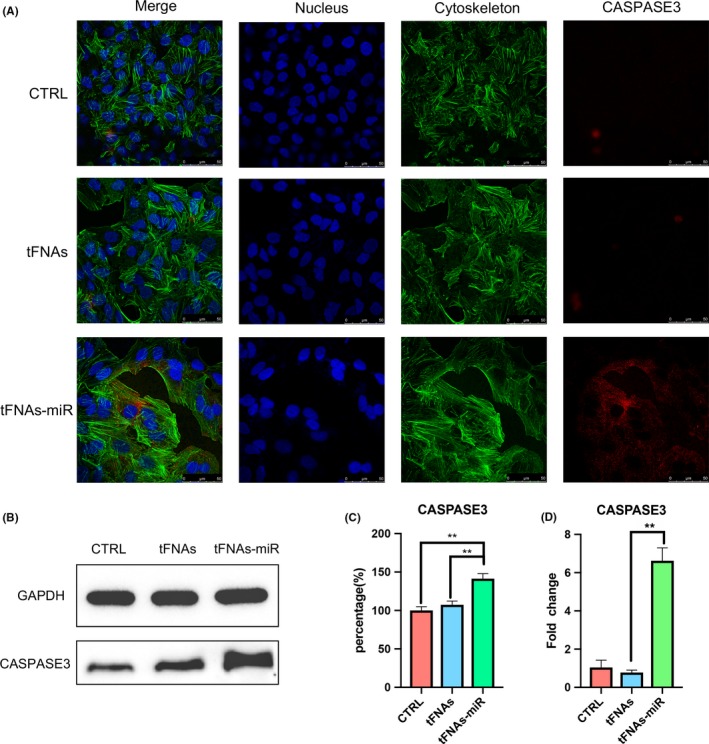
The expression of pro‐apoptotic protein‐Caspase3. A, Immunofluorescence detection of Caspase3 expression after treated with tFNAs and tFNAs‐miR‐214‐3p for 72 h. (nucleus: blue; cytoskeleton: green; Caspase3: red). Scale bars are 50 μm. B, Western blot detection of Caspase3. C, Quantification of Western blot. Data are presented as mean ± standard deviation (SD) (n = 4). Statistical analysis: **P < .01. D, qPCR analysis of Caspase3. Data are presented as mean ± standard deviation (SD) (n = 4). Statistical analysis: **P < .01

**Figure 7:**
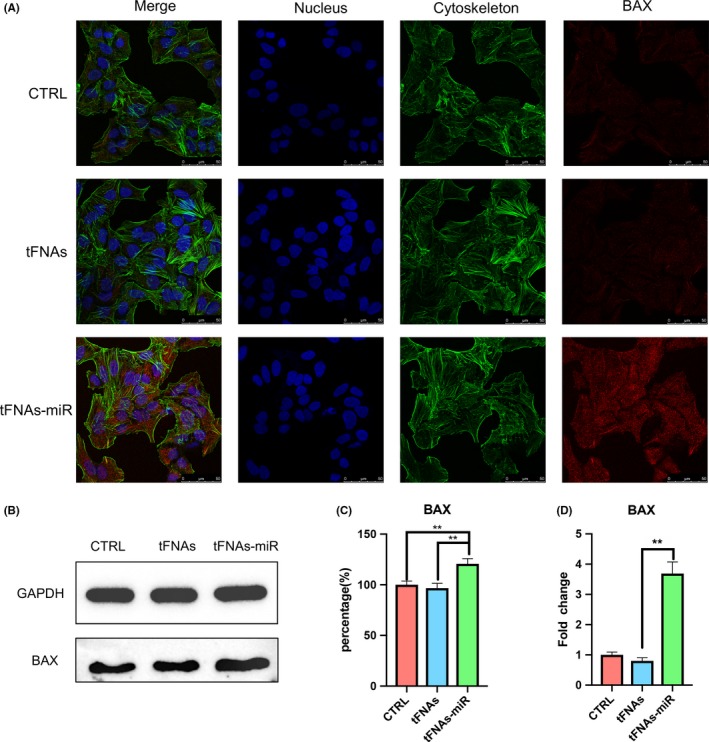
The expression of pro‐apoptotic protein‐Bax. A, Immunofluorescence detection of Bax expression after treated with tFNAs and tFNAs‐miR‐214‐3p for 72 h. (nucleus: blue; cytoskeleton: green; Bax: red). Scale bars are 50 μm. B, Western blot detection of Bax. C, Quantification of Western blot. Data are presented as mean ± standard deviation (SD) (n = 4). Statistical analysis: **P < .01. D, qPCR analysis of Bax. Data are presented as mean ± standard deviation (SD) (n = 4). Statistical analysis: **P < .01

**Figure 8:**
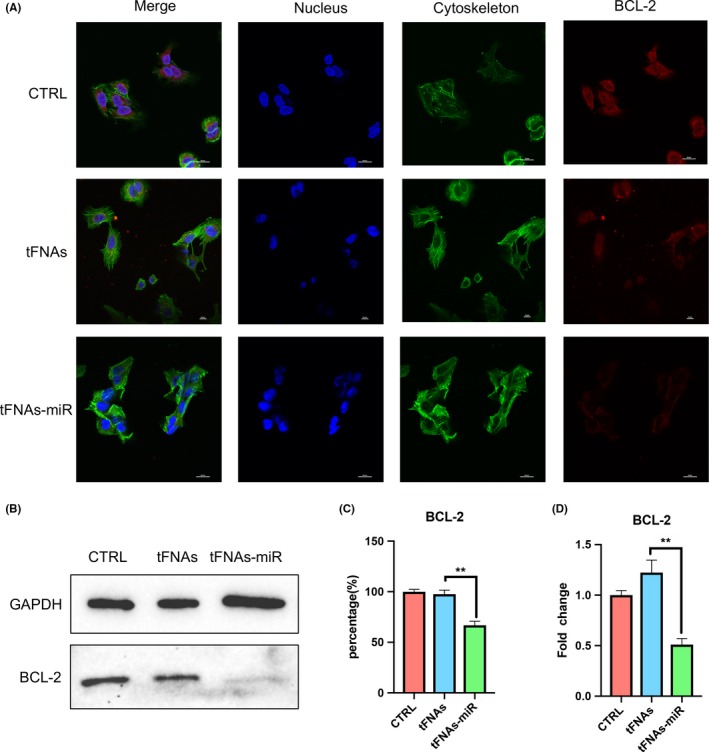
The expression of anti‐apoptotic protein‐Bcl‐2. A, Immunofluorescence detection of Bcl‐2 expression after treated with tFNAs and tFNAs‐miR‐214‐3p for 72 h. (nucleus: blue; cytoskeleton: green; Bcl‐2: red). Scale bars are 50 μm. B, Western blot detection of Bcl‐2. C, Quantification of Western blot. Data are presented as mean ± standard deviation (SD) (n = 4). Statistical analysis: **P < .01. D, qPCR analysis of Bcl‐2. Data are presented as mean ± standard deviation (SD) (n = 4). Statistical analysis: **P < .01

To further verify that tFNAs‐miR‐214‐3p promote apoptosis in A549 cells, gene expression levels (survivin, caspase3, BAX and BCL‐2) were measured by qPCR. In the experimental group, we found that the expression of survivin and BCL‐2 genes decreased significantly by an average of 6.8‐ and 2.4‐fold, respectively (Figures 4D and 8D). Further, the expression of caspase3 and BAX increased significantly by an average 8.5‐ and 4.6‐fold (Figures 6D and 7D).

Immunofluorescence experiments were used to detect three important proteins in the mitochondrial apoptotic pathway, and the intensity of red fluorescence corresponds with the amount of protein expressed. In Figure 6A, we could see that the red fluorescence intensity of the experimental group was the strongest, and the red fluorescence of the tFNAs group and the control group are not detected, which proves that the cells in the experimental group were in apoptotic state. The same trend could also be seen in Figure 7A, demonstrating the high expression of BAX. The weak red fluorescence of BCL‐2 in the experimental group in Figure 8A also confirmed that the cells underwent apoptosis induced by the mitochondrial apoptosis pathway.

From the outcomes of Western blotting, the expression of BCL‐2 protein in the experimental group decreased; further, the expression of caspase3 and BAX was determined to be increased, which was also consistent with the results of qPCR. According to the grey value analysis of the bands of Western blot, it was found that caspase3 and BAX increased by 1.32‐ and 1.25‐fold, respectively (Figure 6B,C and Figure 7B,C), and BCL‐2 decreased to 0.68‐fold (Figure 8B).

## 4 DISCUSSION

In summary, our work has constructed a tFNAs delivery system capable of carrying miRNAs and confirmed that the generated construct could enter A549 cells to interfere with the expression of survivin, which activate the mitochondrial apoptotic pathway and eventually leads to apoptosis. The unique expression of survivin in tumour cells and embryonic cells makes tFNA‐miRNA a new targeted therapy.

Based on the good editability of tFNAs, we can modify more miRNAs at different vertices of tFNAs in the future. There are many microRNAs participate in the process of regulating apoptosis, forming a complex network. Therefore, in the subsequent experiments we will also try to modify the miRNAs acting on the same network on tFNAs, so that these miRNAs can work synergistically. Previous studies have found that tFNAs contact cell membranes with its apex to reduce electrostatic repulsion.^32^ In this study, single‐stranded microRNAs were used for the experiments, and tFNA‐miR contacted the cell membrane may lead to the destruction and degradation of microRNAs before entering the cells. Therefore, we can try double‐stranded microRNAs to transport into the cells in future experiments. Our experiment is only an attempt to study the effect of nanocomposites composed of tFNAs and miRNAs. There are still many aspects that need to be improved and optimized. In addition, due to the functional diversity of miRNAs, this delivery system approach can be applied to a wide variety of fields, such as targeted therapy and tissue regeneration.^41^

## CONFLICT OF INTEREST

No competing interests exist.

## Data Availability

The data that support the findings of this study are available from the corresponding author upon reasonable request.
